# Acute Generalized Exanthematous Pustulosis in an African American Male Caused by Trimethoprim-Sulfamethoxazole

**DOI:** 10.7759/cureus.9591

**Published:** 2020-08-06

**Authors:** Moshe Y Bressler, Jeremy Minkowitz, Naeha Pathak, Andrew Mekaiel, Rebecca Tamez

**Affiliations:** 1 Dermatology, New York Institute of Technology College of Osteopathic Medicine, Old Westbury, USA; 2 Dermatology, Jamaica Hospital Medical Center, Jamaica, USA; 3 Internal Medicine, Jamaica Hospital Medical Center, Jamaica, USA

**Keywords:** agep, bactrim, trimethoprim-sulfamethoxazole, tmp-smx, drug-induced hypersensitivity, human leukocyte antigen (hla), predictive genetic testing, immune system, african american

## Abstract

Acute generalized exanthematous pustulosis (AGEP) is a rare drug-induced autoimmune disease that presents with hundreds of sterile pustules and systemic symptoms. Genetic predisposition, race, and medications prescribed are all factors in AGEP’s frequency, which occurs most commonly in Caucasians and with the use of macrolides and aminopenicillins. Cases of AGEP with sulfonamides or in African American patients are rare. To our knowledge, this is the first documented example of trimethoprim-sulfamethoxazole-induced AGEP in an African American male. In this article, we will further discuss our case and review the literature.

## Introduction

Acute generalized exanthematous pustulosis (AGEP) is a drug-induced T-cell-mediated disease that presents with fever, leukocytosis, and sterile pustules. Rarely, severe AGEP may present with targetoid lesions and mucosal involvement, similar to Stevens-Johnson syndrome and toxic epidermal necrolysis [1]. The mortality rate of AGEP is estimated at 5%. Disease onset is usually between 24 and 48 hours after initiating a previously exposed drug. Treatment of AGEP involves discontinuing the offending medication [2]. Topical steroids are used to relieve the symptoms of AGEP, and intravenous steroids can be used in severe cases [1].

The incidence of AGEP in the United States is five cases per million people, which varies with geographic location and ethnicity [3]. AGEP is more common in females and Caucasians and rare in African Americans [2,3]. A review of the literature revealed only one highly complicated case of AGEP in an African American female who was associated with trimethoprim-sulfamethoxazole (TMP-SMX) as well as several other medications [4]. In this report, we present a unique case of AGEP caused by TMP-SMX in a relatively healthy African American male patient.

## Case presentation

**Figure 1:**
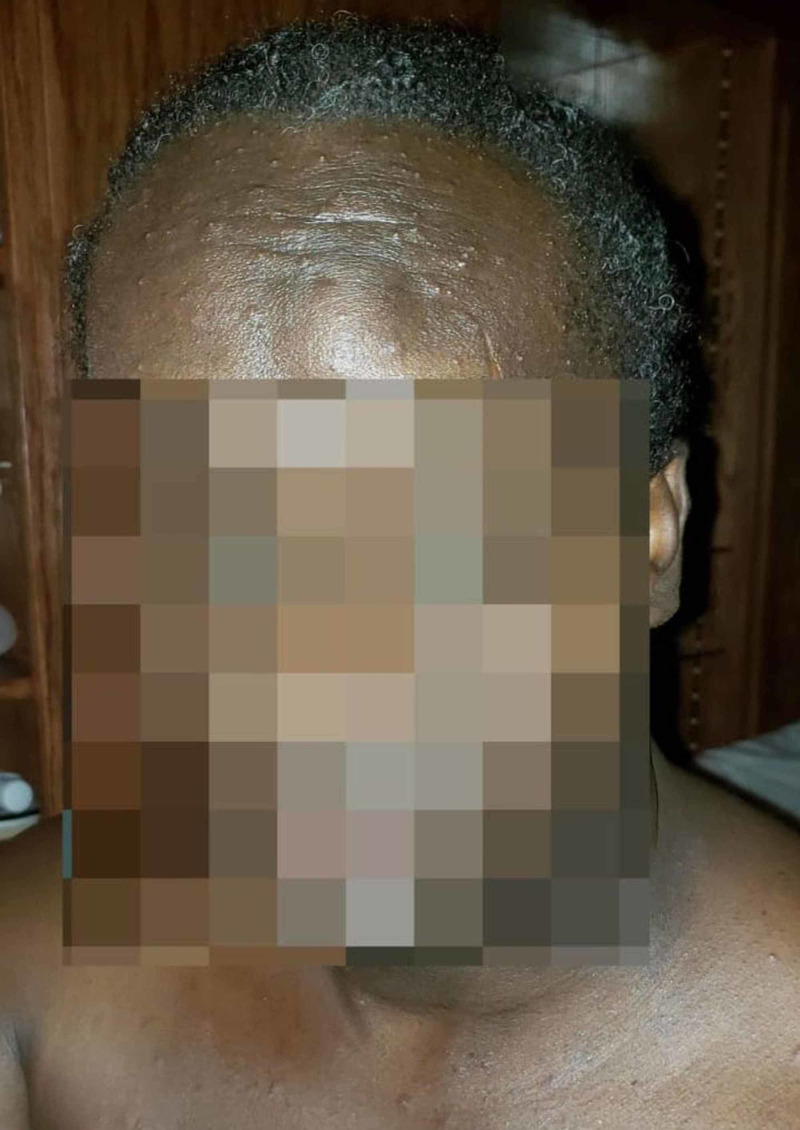
Acute generalized exanthematous pustulosis in a 55-year-old male presenting as sudden vesicle formation and facial swelling four days after beginning trimethoprim-sulfamethoxazole.

**Figure 2:**
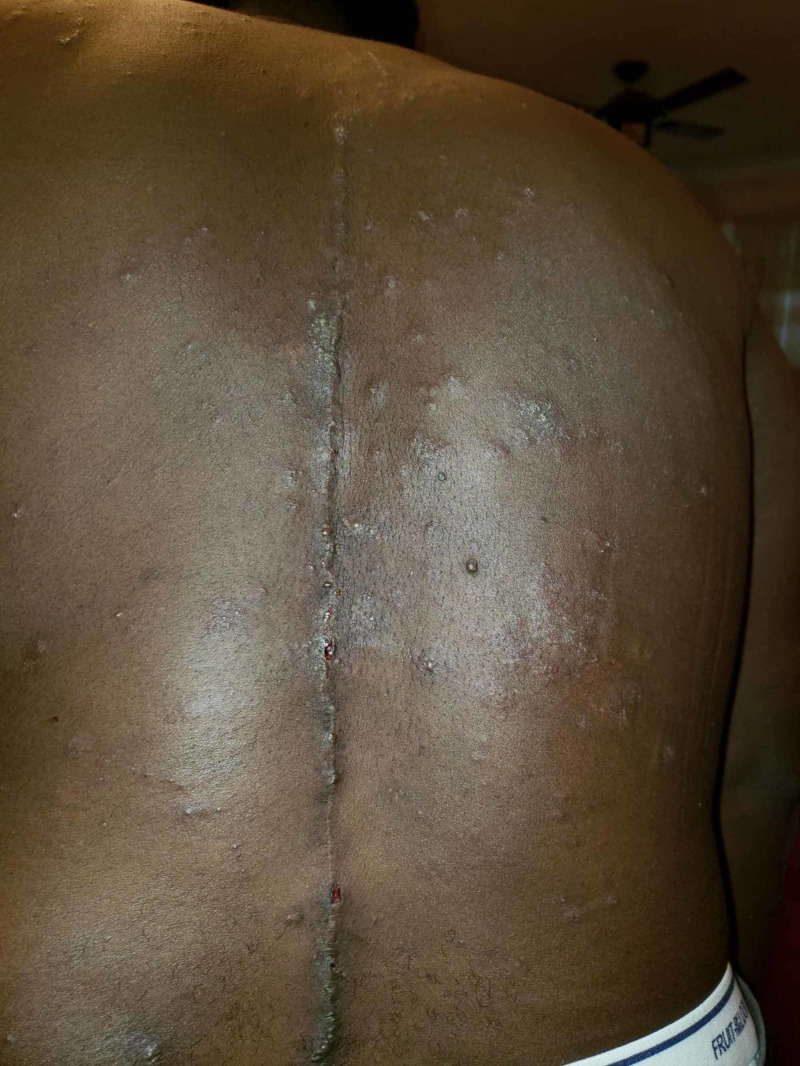
Sterile pustules distributed throughout the back and torso (note previous surgical scar).

**Figure 3:**
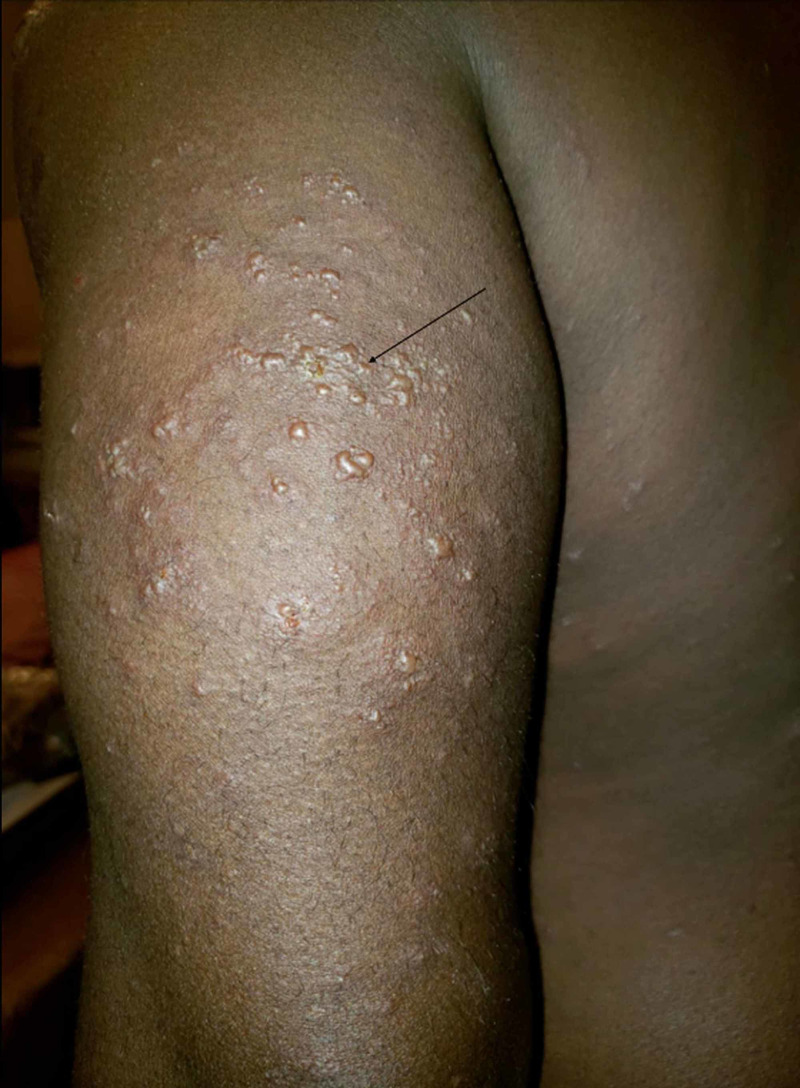
Pustules on an erythematous base beginning to burst and coalesce (black arrow).

A 53-year-old African American male with past medical history of deep venous thrombosis and chronic venous insufficiency presented to the emergency department with a four-day history of fever, malaise, poor appetite, and pruritic pustules over his upper extremities, torso, and back. Eight days before presentation, the patient was prescribed TMP-SMX for presumed lower extremity cellulitis secondary to a venous ulcer. The patient was not on any other medication. On day 4 of treatment, the patient experienced a sudden onset of fever, facial swelling, and rash (Figures 1-3). The patient denied any prior history of skin disease or reactions to medications.

**Figure 4:**
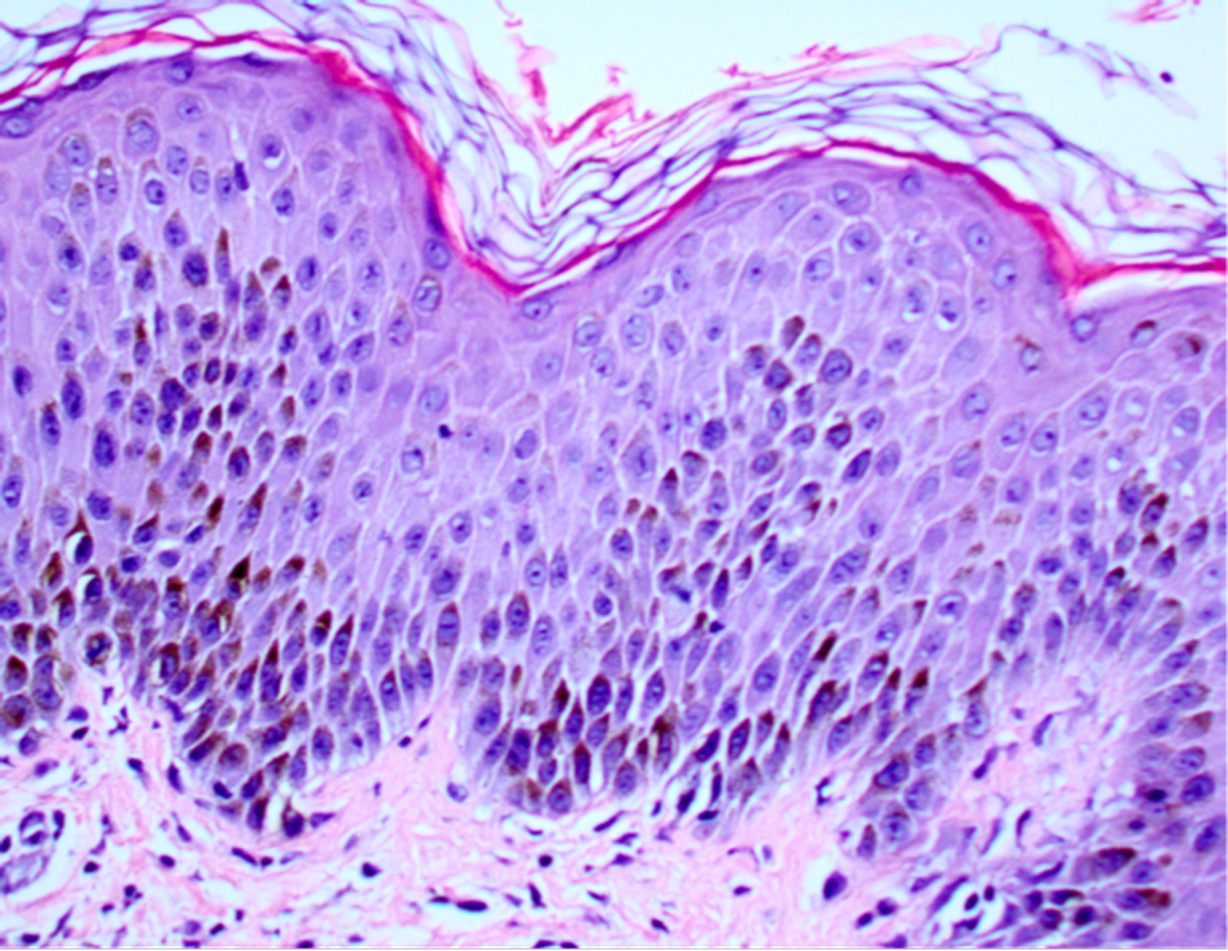
Histology of an unaffected segment of skin taken from a 55-year-old African American male presenting with acute generalized exanthematous pustulosis.

**Figure 5:**
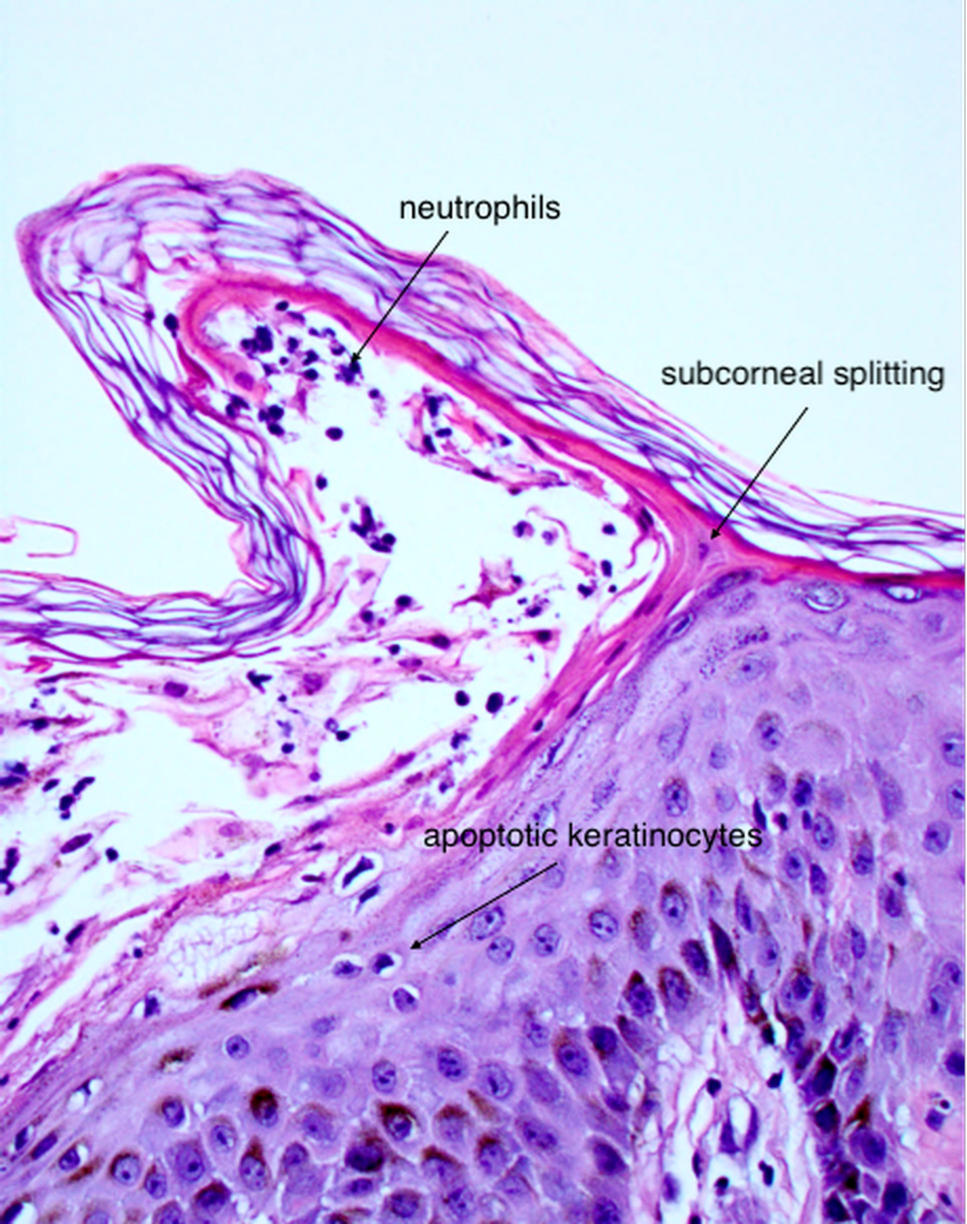
Splitting of the subcorneal layer forming a sterile vesicle with neutrophil infiltration.

**Figure 6:**
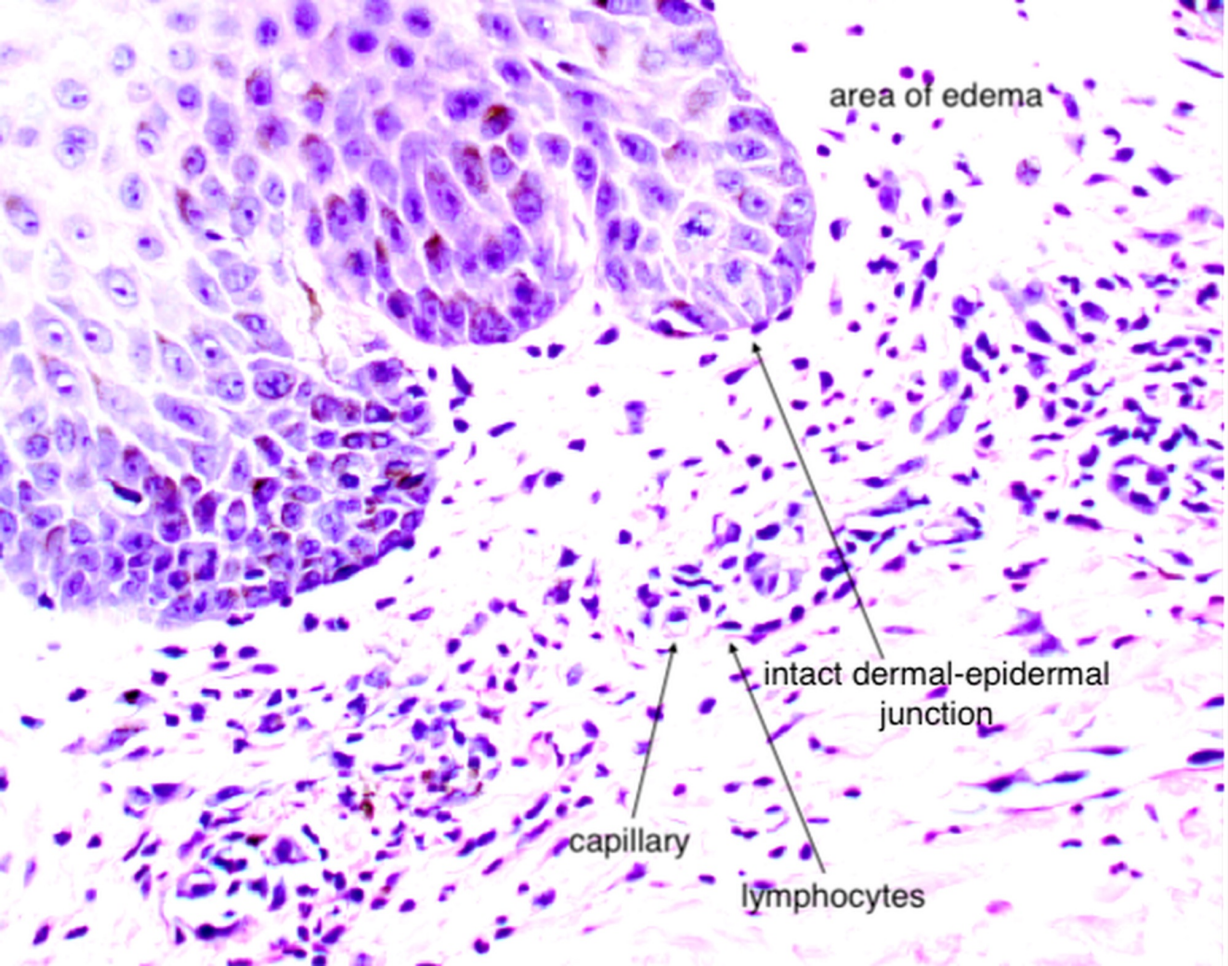
Dermal perivascular lymphocytic infiltration with edema.

**Figure 7:**
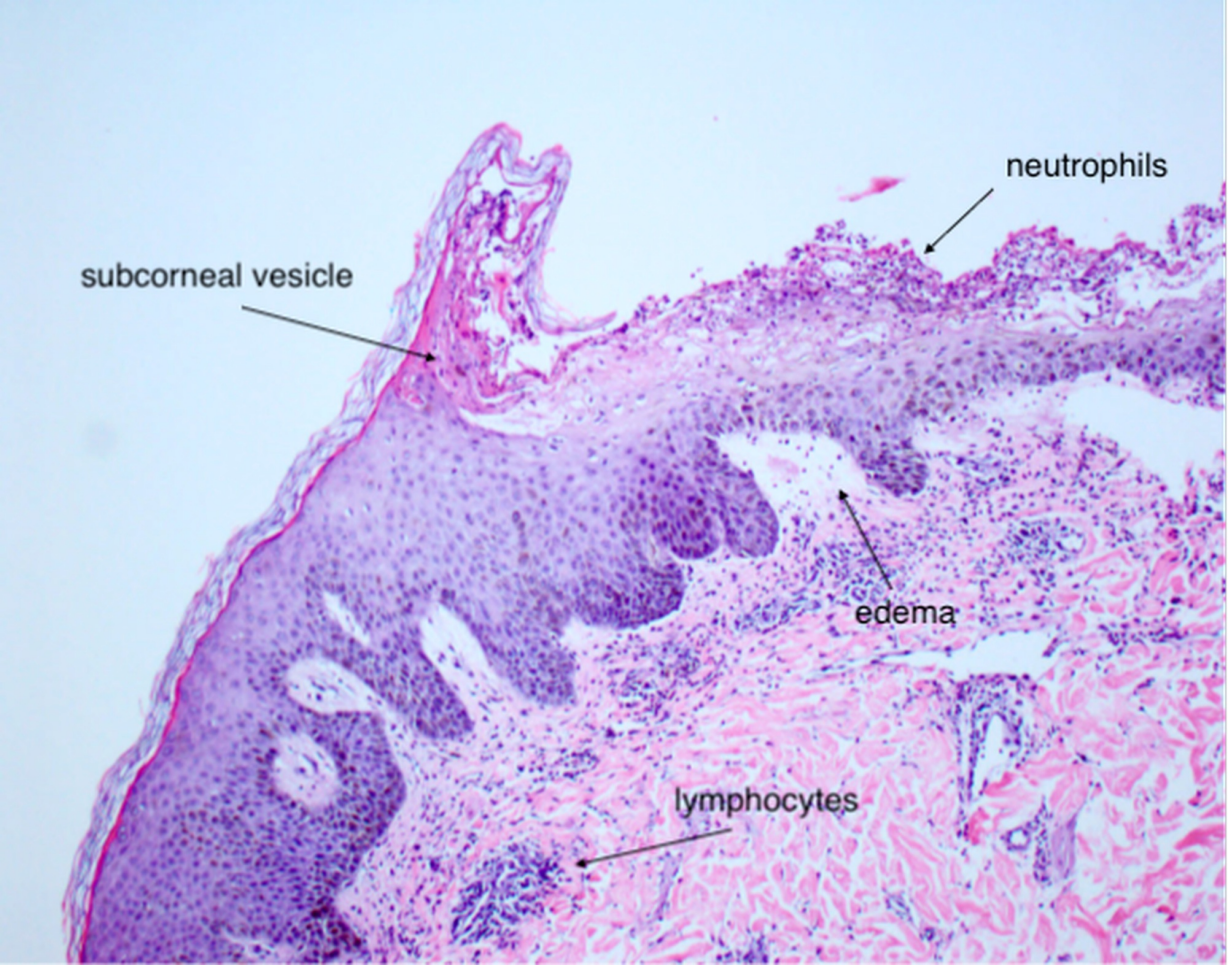
Macro view of dermal lymphocytic infiltration with edema and subcorneal pustule containing neutrophils.

Physical exam showed erythematous plaques with overlying pustules on bilateral arms, thighs, axilla, and back. Vital signs noted a fever of 39.3ºC and tachycardia of 114 bpm. Labs were significant for leukocytosis of 14,400/μL (4,000-11,300/μL), an elevated C-reactive protein of 24.7 mg/L (<10.0 mg/L) and an erythrocyte sedimentation rate of 74 mm/hr (<13 mm/hr). The consulting dermatologist suspected AGEP and confirmed the diagnosis with a skin biopsy (Figures 4-7) and a negative wound culture. The offending agent, TMP-SMX, was discontinued, and topical triamcinolone, 0.1% ointment, was started.

The patient’s constitutional symptoms and respective skin findings began to resolve shortly after discontinuation of the offending medication, and he was subsequently discharged after two days. Follow-up at two weeks showed post-inflammatory hyperpigmentation and complete resolution of other systemic and cutaneous symptoms.

## Discussion

AGEP was a finding first termed in 1980 after being previously misclassified as a drug-induced subtype of general pustular psoriasis [1]. The exanthem’s onset can be up to 15 days post-exposure from a previously sensitized drug, most commonly within the first 24 to 48 hours [1,2]. AGEP is often associated with the use of macrolides, aminopenicillins, and other antibiotic medications [2]. Few reports suggest that some viral and bacterial infections are also an etiology of AGEP [1]. However, European case-control surveillance of severe cutaneous adverse reactions (EuroSCAR) studies concluded that these infections led to antibiotic use, and the medications are ultimately responsible for the disease presentation [2].

AGEP is a type IV hypersensitivity reaction to medications. Previous research has found that drugs and drug metabolites stimulate cytotoxic CD8^+^ T cells, or CD4^+^ T helper cells initiate autoimmune reactions that attack self-tissue [5]. These, in turn, release cytotoxic proteins, causing apoptosis of keratinocytes, forming the pathognomonic sterile vesicles and pustules. Cytokine responses often trigger other systemic symptoms, such as fever, leukocytosis, and occasional eosinophilia. 

The cutaneous manifestations of AGEP present as hundreds of small sterile pruritic pustules that favor the trunk and intertriginous regions, but can spread to anywhere throughout the body. Edema is common, most notably present on the face. A systemic inflammatory response leads to fever, leukocytosis, and internal organ involvement in approximately 17% of cases [1].

The diagnosis of AGEP can be made with clinical presentation, history of exposure, and negative wound cultures. The gold standard for diagnosis is with biopsy confirmation [1]. Histopathology confirmation from an active lesion site will show spongiform subcorneal pustules, edematous papillary dermis with perivascular infiltrates, necrotic keratinocytes, and leukocytoclastic vasculitis with neutrophils and eosinophils [5].

It is crucial in the treatment of AGEP to have a low clinical threshold of suspicion and stop the offending medication once the disease is presumed. Potent topical steroids are given to reduce pruritus, and antiseptic solutions and moist dressings are applied to active lesions to prevent superinfections [1]. Systemic steroids are used in severe cases\. Admission to an ICU is warranted when necessary.

Some genetic populations may be at increased risk of developing AGEP. There are known familial mutations in the anti-inflammatory IL36RN gene that predispose patients to develop AGEP [1]. Additionally, there is an association between sulfamethoxazole-induced severe skin reactions and the human leukocyte antigen (HLA)-B*38 genotype, commonly found in Caucasians [6]. A recent study found that Caucasians experienced a fourfold incidence in AGEP compared to African Americans, perhaps corresponding to the increased genetic diversity in drug metabolism-related genes seen in African Americans, which decreases the frequency of disease-causing alleles [3,7]. Patients with a personal or family history of severe reactions to medications can have genetic testing for IL36RN and HLA-B*38.

A literature search using PubMed and OVID with the terms “AGEP,” “acute generalized exanthemous pustulosis,” and “TMP-SMX,” “trimethoprim,” or “sulfamethoxazole” from the years 1990-2020 returned 10 separate cases: most involved polypharmacy (9/10), 1/10 was not biopsy-confirmed, and 4/10 were immunocompromised with complicated disease courses including: chemotherapy for treatment of glioblastoma, Burkitt’s lymphoma, chronic myelogenous leukemia status post stem cell transplant, and AIDS with a disseminated multidrug-resistant tuberculosis infection [4,8-16].

A literature search using PubMed and OVID of the terms “AGEP,” “acute generalized exanthemous pustulosis,” and “African American,” “black,” and “African” for AGEP and African Americans returned six cases. Most of the cases involved polypharmacy (4/6) or an immunosuppressed state (4/6) [4,17-20].

To the best of our findings, our case is the first occurrence of AGEP caused by TMP-SMX in an African American with no systemic illnesses or polypharmacy.

## Conclusions

AGEP is a rare cutaneous disease that ranges from relatively benign to severe systemic involvement requiring hospitalization. The genetic predisposition to develop AGEP has not been explored in great detail; however, there is some evidence that links the Caucasian population at a higher risk of developing the disease. In the future, as more AGEP cases present and clinicians are more aware of the disease entity, we may see similar cases in minority and underrepresented populations. Familiarity with the illness is a significant key to diagnosing AGEP, and documentation of its presentation is essential to increasing awareness.
